# A case study of soil food web components affected by *Fallopia japonica* (Polygonaceae) in three natural habitats in Central Europe

**DOI:** 10.21307/jofnem-2019-042

**Published:** 2019-07-23

**Authors:** Andrea Čerevková, Lenka Bobuľská, Dana Miklisová, Marek Renčo

**Affiliations:** 1 Institute of Parasitology, Slovak Academy of Science, Košice, Slovakia; 2 Department of Ecology, Faculty of Humanities and Natural Sciences, University of Prešov, Slovakia.

**Keywords:** *Fallopia japonica*, Soil physical properties, Microbial activity, Soil nematode communities, Ecology

## Abstract

This study determined the effect of the invasive plant *Fallopia japonica* on soil physical properties, microbial respiration, microbial biomass carbon content, enzymatic activities, and soil nematode communities. We established in total 30 plots in three natural habitats (forest, grassland, wetland) that were either uninvaded or mostly monospecifically invaded by *F. japonica*. The soil physical and microbial properties differed among the investigated plots, but the differences were observed to be non-significant between the invaded and the uninvaded plots. Non-metric multidimensional scaling based on nematode species diversity indicated that the total number of identified nematode species and their abundance were higher in the uninvaded compare to the invaded plots. Negative effect of *F. japonica* on omnivores, plant parasites, and root-fungal feeder nematodes was confirmed by their lower abundance in the invaded compared to the uninvaded plots. In the invaded plots, we also confirmed lower Maturity and Channel index, but higher Enrichment index. Our results thus indicated that the invasive plant *F. japonica* could affect nematode communities, more than physical or microbial properties, regardless of habitat.

The impact of invasive plants on soil ecosystems in the last decades has attracted world-wide attention. Exotic plant invasions often have dramatic impacts on the resident vegetation by modifying its composition and structure (Levine et al., 2003). Invasive plants have been reported to alter abiotic properties (Rahmonov et al., 2014; Suseela et al. 2016; Stefanowicz et al., 2017), nutrient availability, organic carbon content (Bardon et al., 2014), soil microbiota (Scharfy et al., 2010; Coats and Rumpho, 2014), and soil mesofauna (Quist et al., 2014; Sterzyńska et al., 2017), with special references to variability and composition of arthropods (Moroń et al., 2009; Lenda et al., 2013; Baranová et al., 2014).

The clonal Japanese knotweed, *Fallopia japonica* (Houtt.) Ronse Decr., is considered to be one of the 100 worst invasive alien species in the world (Lowe et al., 2000). It was introduced into North America and Europe in the nineteenth century as an ornamental plant (Bailey and Conolly, 2000) and cattle fodder (Beerling et al., 1994). *F. japonica* produces a large amount of biomass and form monospecific stands that can have a major impact on ecosystem functions (Mincheva et al., 2014) and on soil biodiversity (Beerling et al., 1994; Muller, 2004). Most of the previous studies on *F. japonica* have focused on methods for their control and eradication (Kabat et al., 2006), effects on natural plants (Aguilera et al., 2010), changes in soil chemical properties (Dassonville et al., 2007), allelopathic effects in experimental conditions (Dommanget et al., 2014), or impact on invertebrate species richness (Beerling and Dawah, 1993; Gerber et al., 2008), but the responses of soil microbial or nematode communities on invasion by *Fallopia* spp. are largely understudied. Dassonville et al. (2010) found that *Fallopia* spp. decreased potential denitrification enzyme activity by reducing soil moisture, denitrifying bacteria density in the soil and potential ammonia and nitrite oxidizing bacteria enzyme activities. *Fallopia* spp. have also been shown to produce antimicrobial and antifungal substances (Kim et al., 2005; Kumaga et al., 2005) that could affect the soil bacterial community.

Soil nematodes are an important group of soil biota, constituting an essential trophic link between primary decomposers, such as soil microflora, and larger animals and are recognized as useful bioindicators of soil conditions (Ritz et al., 2009) due to their abundance, diversity, and trophic structure (Bongers, 1990; Yeates et al., 2009). Root tissues and soil microorganisms such as bacteria and fungi represent a primary energy sources for nematode communities, and the quantitative variation of these resources may affect the structural and trophic diversity of nematode communities (Biederman and Boutton, 2009; Ciobanu et al., 2015). Different ecosystems have specific compositions of soil microbial and nematode communities. Estimating the status of and related changes in the structures of microbes and soil nematode communities after the establishment of the invasive plant *F. japonica* must thus include the assessment of different habitats (Renčo and Baležentiené, 2011). For this case study, we chose three habitats (forest, grassland, and wetland) and adjacent territories invaded by *F. japonica* in a valley in Central Europe (Slovakia) to confirm or reverse the effect of *F. japonica* on selected food web components.

To our knowledge, this is the first study observing the impact of *F. japonica* on the soil microbial and nematode community structure. Our objectives were to (i) determine the impact of *F. japonica* on soil pH and moisture, soil microbial respiration, soil microbial biomass carbon content, enzymatic activities and (ii) compare the abundance, diversity, trophic structure of nematode communities and selected ecological indices in three different habitats in *F. japonica*, the invaded and the uninvaded plots. The hypothesis tested was that soil physical properties, microbial, and nematode communities change when the ecosystem is disrupted by the invasion.

## Material and methods

### Study area

The experiment was conducted in a valley near the village of Opátka in South Eastern Slovakia, Central Europe. This region has a temperate climate, with an annual average of 40 summer days per year and a warm, moderately dry sub-region with a mild winter. The average daily temperature in January ranges from 1.5 to 4.0°C, the average daily temperature in July ranges from 16.0 to 18.5°C, while the average annual temperature ranges from 5.0 to 7.0°C. The mean annual precipitation is 650 to 700 mm. The soils are characterized as Fluvisols, and the vegetation zone is characterized as Carpathian oak-hornbeam forest. The landscape is patchy, with deeply undulating uplands (Miklós, 2002). The first *F. japonica* specimens appeared in the village of Opátka around 1992 (personal communication with forester of the cadastre). It was later probably transferred to the entire valley (7 km) below the village, invaded the banks of the creek and then spread to adjacent habitats thus creating large monocultures.

For studying the impact of *F. japonica* on soil physical properties, microbial, and soil nematode communities, we selected three habitats in the valley, namely, forest, grassland, and wetland and corresponding areas adjacent to them invaded by *F. japonica*.

Forest (F) (48°47.63′N, 21°03.43′E; 455 m a.s.l.): covered by a natural, undisturbed, 100 years old deciduous Querco-Fagetea forest, mainly consisting of *Quercus robur*, *Q. cerris*, *Carpinus betulus*, *Acer campestre*, and many shrub species such as mostly *Viburnum* sp. and *Prunus spinosa*.

Forest edge invaded by *F. japonica* (FF): a nearly monospecific stand of *F. japonica* covering an area of 500 m^2^, with an estimated time of invasion of 15 years.

Grassland (G) (48°48.14′N, 21°03.40′E; 392 m a.s.l.): covered with indigenous multispecies vegetation dominated by *Dactylis glomerata*, *Lolium perenne*, *Trifolium pratense*, and *Achillea millefolium*; irregularly mown.

Grassland edge invaded by *F. japonica* (GF): an adjacent of *F. japonica* covering an area of 250 m^2^, with an estimated time of invasion of 15 years.

Wetland (W) (48°48.34′N, 21°03.35′E; 386 m a.s.l.): covered by *Petasites hybridus*, *Caltha palustris*, *Galium aparine*, *Equisetum* sp., *Ranunculus* sp., and *Urtica* sp.; the soil is regularly flooded mostly in the spring, and the vegetation is mown once a year in the autumn.

Wetland edge invaded by *F. japonica* (WF): an adjacent area of 200 m^2^, with an estimated time of invasion of 10 years, the soil is regularly flooded mostly in the spring.

We selected a 25 m × 25 m area of the three different habitats (F, G, and W) which was not yet colonized by *F. japonica*. The distance between F, G, and W along the valley was approximately 1,500 m. A pair of the invaded and the uninvaded areas which did not differ in elevation, inclination, exposition, or management were chosen and the distance between the invaded and the uninvaded areas was 50 m. In each invaded area, we installed five randomly chosen 1 × 1 m plots (approx. 10 m apart) which had similar cover of *F. japonica*. Similar, five 1 × 1 m plots with random distribution were installed in corresponding uninvaded F, G, and W areas. This resulted in 30 plots (five plots × two invasion state [invaded and uninvaded] × three habitats [F, G, and W]). The uninvaded areas were assumed to represent the situation prior to the invasion of *F. japonica*.

### Sampling procedure

The soils were sampled using a garden trowel to depths of 0 to 20 cm in May 2016. A quadrat sampling method was used. Five soil subsamples were collected from each quadrat (1 m^2^), one from each corner and one from the center. The subsamples from each quadrat were then bulked to obtain five representative soil samples (1 kg) for each area. The soil samples were transferred to the laboratory in plastic bags. The bags were stored at 5°C until processing (storage time of soil samples were no longer than one week). Each sample was gently homogenized manually before processing

### Soil physical properties

Soil pH was determined for air-dried soil samples in a 1:3 solution of soil: 0.01 M CaCl_2_ using a pH meter inoLab pH 720-WTW GmbH, Weilheim, Germany. Soil moisture content was measured gravimetrically after the soil had been dried to a constant weight in an oven at 105°C for 24 hr. All determinations were performed in triplicate.

### Soil microbial properties

Soil microbial respiration (SMR) was measured by the amount of CO_2_ released from 100 g of field-moist soil and absorbed by NaOH (μg C-CO_2_/g soil) in hermetically sealed bottles (Alef and Nannipieri, 1995) at 25°C for 24 hr. Microbial biomass carbon (MBC) content was determined using the method of Islam and Weil (1998), as oven-dried equivalent (ODE) of field-moist soil adjusted to 80% water-filled porosity was irradiated twice by microwave (MW) energy at 400 J g^-1^ ODE soil to kill the microorganisms. The time settings and MW oven power depended on the total amount of soil in the MW oven. After cooling, soil samples were extracted with 0.5 M K_2_SO_4_. Carbon content (C_irradiated_) in the extract was quantified by the oxidation with K_2_Cr_2_O_7_ dissolved in H_2_SO_4_ and titrimetrically by (NH_4_)_2_Fe(SO_4_)_2_. The same procedure was done with a non-irradiated sample (C_non-irradiated_). The microbial biomass carbon was then determined as MBC = (C_irradiated_-C_non-irradiated_)/K_ME,_ whereby extraction efficiency factor K_ME_ = 0.213. The activities of acid and alkaline phosphatase were determined by the modified method of Grejtovský (1991) using p-nitrophenyl phosphate as a substrate with incubation at 37°C for 24 hr. Urease activity was determined using urea as a substrate with incubation at 37°C for 3 hr as described by Chazijev (1976), and invertase activity was determined using sucrose as a substrate with incubation at 37°C for 24 hr as described by Schinner and Vonmersi (1990). The control measurements for enzymatic activity did not use the substrate. The activity of all enzymes was measured spectrophotometrically by create a reference curve.

### Nematode extraction and identification

Nematodes were isolated from 100 g of the mixed fresh soil samples by a combination of Cobb sieving and decanting (Cobb, 1918) and a modified Baermann techniques (Van Bezooijen, 2006). Nematodes were extracted from aqueous soil suspensions using a set of two cotton-propylene filters. Subsamples were removed after extraction for 48 hr at room temperature. The aqueous suspensions containing nematodes were examined under a stereomicroscope, excessive water was removed, and the nematodes were fixed in hot fixative 99:1 solution of 4% formaldehyde: pure glycerol and evaluated on permanent glycerine slides (Southey, 1986). All isolated nematodes were microscopically examined at 100, 200, 400, 600, and 1,000 × magnification, identified from permanent glycerine slides mostly to species level (juveniles were identified to genus level) using an Eclipse 90i Nikon, Japan light microscope, with original species descriptions, and several taxonomic keys: Brzeski (1998), Loof (1999), Siddiqi (2000), Andrássy (2005, 2007, 2009), and Geraert (2008, 2010).

Cysts of *Heterodera* juveniles were extracted by floatation (Sabová and Valocká, 1980) from 100 g of soil for species identification based on morphological markers and morphometric data for both cysts and juveniles.

### Nematode community analysis and ecological and functional indices

Nematode species were assigned to trophic groups: bacterivores, fungivores, omnivores, predators, plant parasites, root-fungal feeders, and insect parasites, according to Yeates et al. (1993) and Wasilewska (1997).

The total number of species, total nematode abundance, mean number of nematodes per trophic group, and the Shannon and Weaver species diversity index (H’spp.) (Shannon and Weaver, 1949) were determined. Basic ecological indices were used to assess the status of the soil habitats using nematode communities. The maturity index (MI) for free-living taxa and the plant parasite index (PPI) for plant-parasitic taxa (Bongers, 1990), the enrichment (EI), structural (SI), channel (CI) (Ferris et al., 2001), and basal (BI) indices (Berkelmans et al., 2003) were calculated using the online program ‘NINJA: An automated calculation system for nematode-based biological monitoring’ (Sieriebriennikov et al., 2014; http://spark.rstudio.com/bsierieb/ninja).

### Statistical analysis

The differences in nematode characteristics (total nematode abundance, abundance of nematodes per trophic group, and species diversity) and basic ecological characteristics (MI, PPI, EI, SI, CI, and BI) were analyzed with two-way ANOVA with ‘ecosystems’ (F, G, and W), ‘invasion status’ (invaded, uninvaded), and their interactions as factors. To meet the assumptions of these parametric tests, Box-Cox transformation was applied with the maximum likelihood approach and Golden Search iterative procedure on. If there was an interaction between ‘ecosystem’ and ‘invasion status’ (total nematode abundance and mean number of bacterivores), post hoc Fisher LSD test was applied separately for each ecosystem to determine the effect of ‘invasion status’. Otherwise, main factor ANOVA with two factors (without interaction) was applied. Consequently, in the case of confirmed significance of ‘ecosystem’, post hoc Fisher LSD test was used.

As untransformed soil physical and microbial properties (pH, SMR, MBC content, and enzymatic activities) were not normally distributed (Shapiro–Wilk test) and transformation did not improve normality, nonparametric statistics were applied. Differences among six combinations of ‘ecosystem’ and ‘invasion status’ were tested separately with Kruskal–Wallis ANOVA, followed by a post hoc multiple comparisons.

The above mentioned statistical analysis were performed using Statistica Cz, version 12.0 (Statsoft, Inc., 2013) and significance of all tests was determined at *p*<0.05, 0.01, and 0.001.

Relationships between plots, nematodes, and selected environmental characteristics (soil pH and soil microbial respiration as constrained variables) were analyzed by ordination techniques. Redundancy analysis (RDA) was performed using Canoco 5 (Ter Braak and Šmilauer, 2012), because response data were compositional and had a gradient of 1.7 standard deviations. The significance of the axis was tested by a Monte Carlo permutation test.

Non-metric multidimensional scaling (NMS) ordination was used to examine any changes in the structure of nematode community for the invaded and the uninvaded habitats. A three-dimensional solution was executed by Autopilot, with the slow and thorough mode and Sørensen (Bray-Curtis) distance (recommended for community data). PC-ORD (McCune and Grace, 2002; McCune and Mefford, 2011) was used for the NMS analysis.

## Results

### Soil physical properties

The soil physical properties, soil moisture, and pH differed substantially (Kruskal–Wallis statistics with *p* < 0.001) among the investigated plots (Table 1). The soil moisture content varied from 9.1% in F to 12.4% in W and from 11.7% in GF to 21.5% in WF. Multiple post hoc comparisons confirmed significantly (*p* < 0.05) higher soil moisture only in the invaded FF than the adjacent uninvaded F. The pH varied from 5.2 in F to 6.8 in W and from 6.4 in FF to 7.2 in WF, but no significant differences were observed between the invaded and the uninvaded plots.

**Table 1.** Means and standard errors (SD) of the soil physical properties, microbial respiration, microbial biomass carbon, and enzymes in different ecosystems: forest F; forest with *F. japonica* FF; grassland G; grassland with *F. japonica* GF; wetland W; wetland with *F. japonica* WF.

		F			FF			G			GF			W			WF		
Soil Indices	H	Mean	SD		Mean	SD		Mean	SD		Mean	SD		Mean	SD		Mean	SD	
Soil moisture (%)	21.93***	9.1	0.04	c	14.0	0.55	ab	9.5	0.35	ac	11.7	0.06	abc	12.4	0.94	abc	21.5	0.98	b
Soil pH (CaCl_2_)	22.30***	5.2	0.06	a	6.4	0.06	ab	6.1	0.06	ac	6.8	0.06	ab	6.8	0.05	bc	7.2	0.06	b
Soil microbial respiration	21.86***	139.6	7.37	abc	185.1	8.14	bc	58.0	14.67	a	123.5	7.28	abc	90.4	15.30	ad	147.8	14.84	bcd
Microbial biomass carbon	17.18**	344.0	33.52	ab	370.6	11.99	a	289.9	10.93	b	297.5	12.6	b	309.5	8.59	ab	351.0	16.15	ab
Urease	35.15***,^1^	1.5	0.03	a	1.3	0.02	ab	1.2	0.11	ab	1.4	0.08	a	0.9	0.26	b	0.6	0.04	b
Acid phosphatase	21.79***	60.0	1.43	a	53.1	2.78	ab	58.2	0.75	ac	57.5	1.00	ab	34.1	4.24	bc	24.1	1.54	b
Alkaline phosphatase	21.37***	26.1	0.65	b	38.3	1.18	ab	31.7	4.57	ab	44.2	1.31	a	34.4	2.23	ab	26.2	0.73	b
Invertase	27.80***,^2^	39.9	1.13	c	33.9	2.91	ac	21.2	0.99	b	25.5	0.77	ab	31.1	1.20	ab	28.2	0.28	ab

Note: H means statistics H (5, N = 24) from Kruskal–Wallis test (For ^1^N = 42 and for ^2^N = 30). Significant differences from multiple post hoc comparisons of site characteristics between ecosystems are indicated by different lowercase letters (a,b,c,d) in every row with p less than 0.05. Significance level: ***0.001; **0.001 levels, respectively.

### Soil microbial respiration (SMR), microbial biomass carbon (MBC) content, and enzymatic activities

SMR and MBC content were higher (but not significantly) in all plots with *F. japonica* (FF, GF, WF) than in the uninvaded plots (F, G, W) (Table 1). Opposite trends were found for acid phosphatase; it was lower (but not significantly) in the invaded (FF, GF, WF) than the uninvaded plots (F, G, W). Urease, alkaline phosphatase, and invertase enzymes were influenced by *F. japonica* invasion in different ways in the different ecosystems, but no significant differences were found.

### Nematode community analysis

A total of 9,452 individual nematodes were isolated and identified. The total number of species and genera of soil free-living and plant-parasitic nematodes was higher in the uninvaded (58 and 46) than in the invaded plots (49 and 40). The species found in the uninvaded (F, G and W) but absent in the invaded plots were as follows: six bacterivores *Acrobeles cylindricus*, *Eucephalobus oxyuroides*, *Mesorhabditis* sp. juv., *Plectus tenuis*, *Prismatolaimus dolichurus*, and *Punctodora* sp. juv.; one fungivore *Tylencholaimus stecki*; one omnivore *Aporcelaimellus obtusicaudatus*; two predators *Coomansus parvus* and *Thonus ettersbergensis*; two plant parasites *Heterodera hordecalis* and *Meloidogyne hapla*; and two root-fungal feeders *Boleodorus thylactus* and *Coslenchus costatus*. On the other hand, only the invaded plots (FF, GF, WF) contained the omnivore *Axonchium coronatum* (GF), the predators *Coomansus zschokkei* (GF) and *Trischistoma monohystera* (WF), and the plant parasites *Hemicycliophora typica* (WF) and *Heterodera* sp. 1 (FF) (Table 2). Mean nematode abundance was significantly higher in the uninvaded compare to the invaded plots (*p* < 0.001). Subsequent post hoc Fisher LSD test confirmed a significant effect of ‘invasion status’ for F compare to FF and for W compare to WF (both *p*<0.05), but not for G compare to GF. Species diversity index did not differ significantly between the invaded and the uninvaded plots (Table 3).

**Table 2.** List of identified nematode species and their mean abundance in ind./100 g of soil (*n* = 5) and standard errors (SD) in different ecosystems: forest F; forest with *F. japonica* FF; grassland G; grassland with *F. japonica* GF; wetland W; wetland with *F. japonica* WF; c-p value of colonizer–persister nematode species from 1 to 5 according to Bongers (1990).

			F		FF		G		GF		W		WF	
Nematode species/trophic groups	Abbr.	c-p	Mean	SD	Mean	SD	Mean	SD	Mean	SD	Mean	SD	Mean	SD
*Bacterivores*														
*Acrobeles cylindricus* (Ivanova, 1968)	Acyl	2	–	–	–	–	3.6	5.7	–	–	0.2	0.4	–	–
*Acrobeloides nanus* (de Man, 1880)	Anan	2	5.4	5.0	3.0	1.9	2.6	3.4	11.2	9.5	18.4	9.4	5.0	5.5
*Alaimus primitivus* (de Man, 1880)	Apri	4	9.6	2.9	2.2	1.1	2.2	3.2	4.0	3.1	7.0	5.0	0.6	0.9
*Cephalobus persegnis* (Bastian, 1865)	Cper	2	0.8	1.8	–	–	9.6	8.6	1.8	1.8	14.6	14.4	3.2	4.0
*Cervidellus vexilliger* (de Man, 1880)	Cvex	2	3.6	4.3	–	–	–	–	1.4	2.1	–	–	–	–
*Eucephalobus oxyuroides* (de Man, 1876)	Eoxy	2	–	–	–	–	0.8	1.8	–	–	–	–	–	–
*Eucephalobus striatus* (Bastian, 1865)	Estr	2	–	–	1.0	1.4	6.4	4.8	0.4	0.9	6.8	5.2	6.2	7.1
*Chiloplacus propinquus* (de Man, 1921)	Cpro	2	0.6	1.3	0.4	0.5	0.2	0.4	1.0	0.7	0.4	0.9	0.2	0.4
*Mesorhabditis* sp. juv.	Msp.	1	0.4	0.9	–	–	–	–	–	–	–	–	–	–
*Panagrolaimus rigidus* (A. Schneider, 1866)	Prig	1	–	–	–	–	–	–	–	–	0.2	0.4	0.8	1.3
*Plectus parietinus* (Bastian, 1865)	Ppar	2	5.0	6.2	0.4	0.9	1.4	2.1	0.2	0.4	4.8	3.6	1.0	1.2
*Plectus parvus* (Bastian, 1865)	Ppai	2	3.8	3.4	1.6	2.5	5.4	3.9	3.0	2.2	7.8	6.1	4.0	3.3
*Plectus tenuis* (Bastian, 1865)	Pten	2	0.2	0.4	–	–	1.2	2.7	–	–	–	–	–	–
*Prismatolaimus dolichurus* (de Man, 1880)	Pdol	3	–	–	–	–	2.8	3.9	–	–	–	–	–	–
*Prismatolaimus intermedius* (Bütschli, 1873)	Pint	3	58.8	59.0	7.8	11.7	4.2	3.3	6.2	4.1	3.6	2.3	1.0	1.2
*Punctodora* sp. juv.	Psp.	3	0.6	1.3	–	–	–	–	–	–	–	–	–	–
*Rhabditis* spp. juv.	Rspp	1	27.8	18.0	39.4	17.3	37.4	24.5	77.6	33.0	146.6	104.5	38.8	26.4
*Teratocephalus terrestris* (Bütschli, 1873)	Tter	3	2.6	2.1	2.6	3.6	1.8	2.7	1.4	2.6	–	–	–	–
*Wilsonema schuurmansstekhoveni* (de Coninck, 1931)	Wsch	2	2.6	4.2	2.2	2.3	0.8	1.3	4.4	4.3	0.6	0.9	1.2	2.2
*Fungivores*														
*Aphelenchoides composticola* (Franklin, 1957)	Acom	2	0.2	0.4	0.6	0.9	2.2	3.2	1.4	2.1	0.6	0.5	–	–
*Aphelenchoides minimus* (Meyl, 1953)	Amin	2	0.2	0.4	–	–	3.6	3.8	0.4	0.5	–	–	–	–
*Aphelenchoides ritzemabosi* (Schwartz, 1911)	Arit	2	–	–	–	–	0.6	1.3	0.2	0.4	3.4	4.6	2.4	1.9
*Aphelenchus avenae* (Bastian, 1865)	Aave	2	–	–	0.6	0.9	6.2	3.9	6.0	6.4	19	29.1	4.0	3.8
*Doryllium zeelandicum* (de Man, 1876)	Dzee	4	–	–	0.2	0.4	4.4	6.2	5.2	9.5	7.6	11.2	1.2	1.8
*Tylencholaimus stecki* (Steiner, 1914)	Tste	4	–	–	–	–	4.0	8.4	–	–	–	–	–	–
*Omnivores*														
*Aporcelaimellus obtusicaudatus* (Bastian, 1865)	Aobt	5	–	–	–	–	0.4	0.9	–	–	–	–	–	–
*Axonchium coronatum* (de Man, 1906)	Acor	5	–	–	–	–	–	–	0.4	0.5	–	–	–	–
*Diphtherophora communis* (de Man, 1880)	Dcom	3	–	–	–	–	8.2	8.8	–	–	26.2	21.9	2.2	2.9
*Enchodelus macrodorus* (de Man, 1880)	Emac	4	1.4	1.9	0.6	1.3	13.6	6.8	–	–	–	–	1.0	1.4
*Eudorylaimus silvaticus* (Brzeski, 1960)	Espp	4	10.8	6.0	1.4	1.7	7.4	2.7	1.2	1.1	9.0	7.0	6.0	4.7
*Eudorylaimus* spp. juv.	Esil	4	7.6	4.3	1.0	1.7	3.6	6.1	–	–	–	–	–	–
*Mesodorylaimus bastiani* (Bütschli, 1873)	Mbas	5	0.8	1.8	0.4	0.9	0.6	0.9	0.6	1.3	9.4	3.5	0.4	0.5
*Microdorylaimus parvus* (de Man, 1880)	Mpar	4	–	–	2.6	2.6	0.8	1.3	11.8	18.2	6.4	5.9	6.8	6.0
*Predators*														
*Anatonchus tridentatus* (de Man, 1876)	Atri	4	0.6	0.9	1.0	1.7	–	–	0.8	1.1	1.0	1.2	1.2	1.8
*Coomansus parvus* (de Man, 1880)	Cpar	4	5.2	3.7	–	–	–	–	–	–	–	–	–	–
*Coomansus zschokkei* (Menzel, 1913)	Czsc	4	–	–	–	–	–	–	1.2	2.2	–	–	–	–
*Mylonchulus brachyuris* (Bütschli, 1873)	Mbra	4	1.2	1.8	2.6	4.0	3.0	2.7	2.2	1.5	3.6	4.4	1.2	1.8
*Tripyla filicaudata* (de Man, 1880)	Tfil	3	5.4	11.0	2.0	4.5	0.2	0.4	–	–	–	–	–	–
*Tripyla setifera* (Bütschli, 1873)	Tset	3	11.2	11.0	7.4	7.8	1.0	1.0	7.4	11.6	–	–	–	–
*Trischistoma monohystera* (de Man, 1880)	Tmon	3	–	–	–	–	–	–	–	–	–	–	2.6	4.7
*Thonus ettersbergensis* (de Man, 1885)	Tett	4	2.0	2.4	–	–	3.0	3.0	–	–	–	–	–	–
*Plant parasites*														
*Bitylenchus dubius* (Bütschli, 1873)	Bdub	3	–	–	12.8	24.8	10.2	4.4	13.2	15.0	17.2	14.8	–	–
*Helicotylenchus digonicus* (Perry, 1959)	Hdig	3	0.8	1.3	11.0	13.2	80.8	51.2	62.2	61.1	69.8	25.9	31.0	25.3
*Helicotylenchus dihystera* (Cobb, 1893)	Hdih	3	–	–	–	–	9.2	5.8	16.0	28.6	2.2	2.9	2.6	3.6
*Hemicycliophora typica* (de Man, 1921)	Htyp	3	–	–	–	–	–	–	–	–	–	–	6.0	11.2
*Heterodera hordecalis* (Andersson, 1975)	Hhor	3	–	–	–	–	1.8	2.5	–	–	–	–	–	–
*Heterodera* sp. 1 juv.	Hsp1	3	–	–	0.8	1.8	–	–	–	–	–	–	–	–
*Heterodera* sp. 2 juv.	Hsp2	3	–	–	–	–	–	–	–	–	1.6		0.4	
*Meloidogyne hapla* (Chitwood, 1949)	Mhap	3	–	–	–	–	1.0		–	–	–	–	–	–
*Mesocriconema curvatum* (Raski, 1952)	Mcur	3	0.2	0.4	–	–	2.4	0.9	–	–	0.2	0.4	0.6	0.5
*Paratylenchus bukowinensis* (Micoletzky, 1922)	Pbuk	2	–	–	2.0	2.3	24.6	22.9	30.6	44.7	–	–	–	–
*Paratylenchus straeleni* (de Coninck, 1931)	Pstr	2	153.0	160.0	0.2	0.4	0.2	0.4	6.4	6.4	28.0	20.2	20.8	33.4
*Pratylenchoides crenicauda* (Winslow, 1958)	Pcre	2	–	–	–	–	5.0	6.4	0.4	0.5	1.6	2.6	–	–
*Pratylenchus pratensis* (de Man, 1880)	Ppra	3	–	–	0.6	0.9	8.4	3.8	0.6	0.9	15.6	24.6	3.4	4.7
*Rotylenchus robustus* (de Man, 1876)	Rrob	3	–	–	4.0	2.1	1.4	3.1	6.8	7.0	3.2	4.1	8.4	6.4
*Trichodorus sparsus* (Szczygiel, 1968)	Tspa	4	33.0	19.0	2.2	4.9	–	–	–	–	14.4	14.0	–	–
*Root-fungal feeders*														
*Aglenchus agricola* (de Man, 1884)	Aagr	2	25.6	45.0	–	–	8.4	7.2	5.0	5.5	0.6	1.3	0.6	1.3
*Boleodorus thylactus* (Thorne, 1941)	Bthy	2	–	–	–	–	19.2	15.6	–	–	–	–	–	–
*Coslenchus costatus* (de Man, 1921)	Ccos	2	5.0	11.0	–	–	1.8	2.7	–	–	–	–	–	–
*Filenchus misellus* (Andrassy, 1958)	Fmis	2	70.2	79.0	–	–	–	–	–	–	14.2	28.0	1.4	1.9
*Filenchus vulgaris*(Brzeski, 1963)	Fvul	2	23.0	29.0	7.2	2.9	20.8	9.1	11.2	12.1	10.0	14.2	6.4	12.7
*Malenchus exiguus* (Massey, 1969)	Mexi	2	7.6	7.1	–	–	5.4	2.7	0.6	1.3	–	–	0.6	1.3
*Insect parasites*														
*Steinernema affine* (Bovien, 1937)	Saff	1	3.0	3.0	–	–	0.4	0.9	0.2	0.4	–	–	–	–
Total number of species			36		31		49		37		35		34	
Total number of genera			30		27		39		32		31		31	

**Table 3.** F-values from two-way ANOVA^d^ or main effect ANOVA with factors ‘Ecosystem’ (forest, grassland, wetland) and ‘Invasion’ (invaded, uninvaded).

							Ecosystem									Invasion					
	Ecosystem		Invasion		Ecosystem×Invasion		Forest			Grassland			Wetland			Uninvaded			Invaded		
Indices	F(1,26)	*p*	F(2,26)	*p*		*p*	Mean	SD		Mean	SD		Mean	SD		Mean	SD		Mean	SD	
Abundance^d^	0.87	ns	25.86	***	5.08	*	305.80	247.48		324.40	117.03		324.50	229.34		436.60	199.02		199.87	111.92	
Species diversity index	3.94	*	3.95	ns			2.09	0.42	a	2.52	0.34	b	2.33	0.31	ab	2.43	0.43		2.19	0.31	
Bacterivores^d^	0.32	ns	5.78	*	4.98	*	92.70	59.04		96.80	45.01		136.50	115.95		138.87	95.54		78.47	44.05	
Fungivores	16.58	***	0.80	ns			0.90	0.88	a	17.10	12.01	b	19.10	27.19	b	17.33	24.05		7.40	8.93	
Omnivores	4.34	*	22.87	***			14.30	12.27	a	25.80	21.46	ab	33.70	27.36	b	37.07	23.29	A	12.13	11.58	B
Predators	2.64	ns	0.27	ns			18.30	12.54		7.90	9.26		4.80	3.94		10.80	11.10		9.87	10.68	
Plant parasites	1.30	ns	11.06	**			110.30	129.87		140.60	82.53		113.50	57.80		161.93	88.30	A	81.00	80.37	B
Root-fungal feeders	4.25	*	9.65	**			69.30	107.53	a	36.20	26.56	a	16.90	31.04	b	70.60	86.71	A	11.00	9.38	B
Maturity Index	0.90	ns	7.05	*			2.22	0.37		2.20	0.32		2.05	0.34		2.31	0.32	A	2.01	0.30	B
Plant-Parasitic Index	2.00	ns	0.15	ns			2.57	0.32		2.58	0.20		2.77	0.19		2.62	0.28		2.66	0.23	
Channel Index	1.18	ns	5.32	*			22.16	29.72		12.44	8.81		8.36	9.38		21.35	24.04	A	7.29	7.91	B
Basal Index	0.65	ns	2.67	ns			10.39	8.76		11.06	5.36		11.22	5.38		12.92	7.23		8.86	5.06	
Enrichment Index	0.09	ns	6.45	*			80.54	16.57		80.95	11.08		83.01	7.97		76.10	13.48	A	86.88	7.42	B
Structure Index	1.27	ns	0.00	ns			78.73	15.51		76.59	11.14		72.35	12.72		76.58	10.65		75.20	15.45	

Note: ^d^There was an interaction between ‘ecosystem’ and ‘invasion status’; df’s are (1, 24), (2, 24) and significant differences among plots from post hoc Fisher LSD test are reported only in text. If significant effect of factor is confirmed by ANOVA, small letters (a, b, c) within ‘ecosystem’ and capital letters A, B within ‘invasion status’ indicate significant differences between sites with p less than 0.05. Means and standard errors (SD) of the nematode community indices and nematode total abundance in the trophic groups in different ecosystems: forest, grassland, wetland; uninvaded and invaded. ^*,**,***^Significant at 0.05, 0.01 and 0.001 levels, respectively.

**Figure 1:**
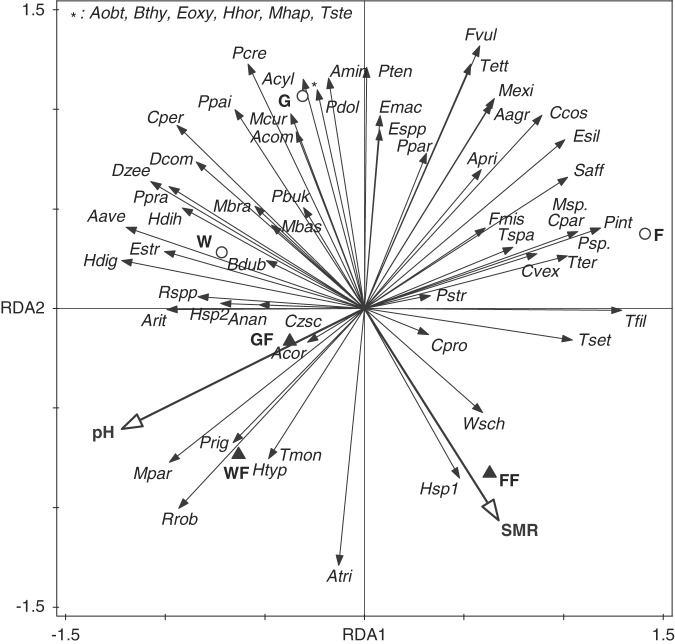
RDA ordination diagram of nematode communities and Soil pH and Soil Microbial Respiration (Resp) as constrained variables in different ecosystems: forest F; forest with *F. japonica* FF; grassland G; grassland with *F. japonica* GF; wetland W; wetland with *F. japonica* WF with 53.5% 500 explained variance (eigenvalues of axis: 0.35; 0.19; pseudo F = 1.7; P = 0.01). For abbreviation of species see Table 2. Empty circles: biotopes without *F*. *japonica*, full triangles: biotopes with *F*. *japonica.*

The ordination analysis identified two explanatory variables (pH and SMR) that accounted for 53.5% of the total variation of nematode species abundance (Fig. 1). Monte Carlo permutation tests identified statistical significance of all axes for this relation (pseudo *F* = 1.7, *p* = 0.01). Soil acidity and SMR were negatively correlated with the occurrence and abundance of most nematode species in F, G, and W, but soil pH positively correlated with the abundance of the predators *A. coronatum* and *C. zschokkei* in GF. The RDA partly separated the nematode communities of the uninvaded habitats from the invaded habitats. *Cephalobus persegnis*, *Plectus parietinus*, *Prismatolaimus intermedius*, and *Mesodorylaimus bastiani* were more abundant in the uninvaded plots, but *Microdorylaimus parvus*, *Anatonchus tridentatus*, and *Rotylenchus robustus* were more abundant in the invaded plots (Fig. 1, Table 2).

**Figure 2:**
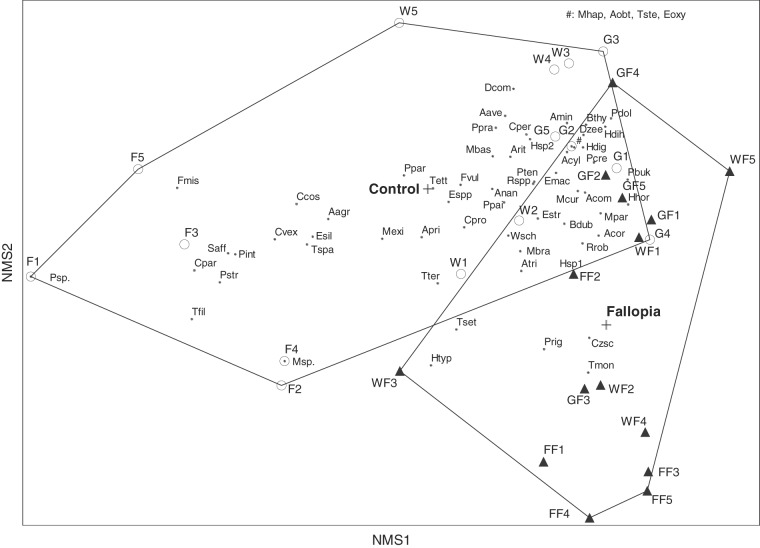
NMS analysis of samples characterized by nematode species abundance, the grouping factor is presence/absence of *F*. *japonica*. Plus signs stand for centroids of two groups, lines around groups connect the outer points of a group. Empty circles represent soil samples from Control: forest F1 to F5, grassland G1 to G5, and wetland W1 to W5. Full triangles represent soil samples with invaded plan *F. japonica*: forest with *F*. *japonica* FF1 to FF5; grassland with *F*. *japonica* GF1 to GF5; wetland with *F*. *japonica* WF1 to WF5. For abbreviation of species see Table 2. Variance explained by axis 1 and 2 is 48 and 28%, respectively.

The NMS analysis compared nematode composition based on species diversity. The best three-dimensional solution for the NMS ordination had a final stress of 10.40 (*p* < 0.0001) after 48 iterations, which was supported by a Monte Carlo permutation tests with a significance of *p* = 0.004 and a mean stress of 10.67 for real data and 250 runs for both real and randomised data. The variances explained by the first and second axes were 48 and 28%, respectively. The NMS analysis identified a notable impact of the invasive plants (Fig. 2). The samples from F, G, and W were clearly separated into one group, and the samples from FF, GF, and WF were separated into another group. Moreover, samples from forest (F) differed the most from the other samples, and were separated to the left in the figure, and were represented mostly by high abundances of *Paratylenchus straeleni, Tripyla filicaudata, Filenchus misellus*, and *Prismatolaimus intermedius.*

### Ecological indices and trophic-group distribution of nematodes

A total of 62 nematode species were identified: 18 of which were bacterial feeders (29.0%), 15 were plant parasites (24.2%), 9 were omnivores (14.5%), 7 were predators (11.3%), 6 were fungivores and root-fungal feeders (both 9.7%), and 1 was an insect parasite (1.6%).

Plant parasites were the most abundant trophic group in F and G (Table 3). The abundance of plant parasites was significantly higher in the uninvaded rather than the invaded plots (*p* < 0.01). Bacterivores were the second most abundant trophic group in F and G and the most abundant trophic group in W. Subsequent post hoc Fisher LSD test confirmed a significantly higher bacterivore abundance (*p* < 0.05) in F compared to FF and in W relative to WF (*p* < 0.05), but not for G and GF was observed. The abundance of fungivores was very low in the forest compared to both the grassland and the wetland (*p* < 0.001), but without significant differences between the invaded and the uninvaded plots. The abundance of root-fungal feeders was significantly higher in the forest and grassland rather than the wetland (*p* < 0.05) and in the uninvaded than the invaded plots (*p* < 0.01). The abundance of omnivores was significantly higher in the uninvaded rather than the invaded plots (*p* < 0.001). No significant differences in the abundance of predators were found.

Maturity and Channel indices were significantly higher in the uninvaded compared to the invaded plots (*p* < 0.05). Structure and Basal indices did not significantly differ between the invaded and the uninvaded plots. When plotting the Enrichment and Structure Indices, most of the soil samples ended up in Quadrat B for both the invaded and the uninvaded plots (data not shown) suggesting that food webs were highly enriched and structured with both bacterial and fungal decomposition channels and maturing food web condition. No significant effect of ‘ecosystem’ was observed in the case of abovementioned nematode indices (Table 3).

## Discussion

The recent exhaustive literature review on Japanese knotweed indicated that invasion may or may not alter chemical properties of the soil, suggesting that impacts depend on the native plant species that the knotweed replace (Lavoie, 2017). Native flora, as well as actual weather conditions, soil and ecosystem type, date of soil sampling, etc., are probably responsible for the variable impacts of invasion on soil acidity and moisture among studies. Dassonville et al. (2011) in Belgium and France and Suseela et al. (2016) in USA found that invasion of *F. japonica* can reduce soil moisture probably due to its great leaf area promoting high transpiration rate. Our results with significant higher soil moisture in the invaded forest FF than in the adjacent uninvaded plots contradict these findings. This result was probably due to shadow of the tree canopy; as we did not observe different soil moisture between the invaded and the uninvaded plots in grassland and wetland. These results are in agreement with those by Stefanowicz et al. (2017) in Poland who found that *F. japonica* invasion did not affect soil moisture in four river valleys. In our study, pH in the invaded plots varied from 6.4 to 7.2, and in the uninvaded plots ranged from 5.2 to 6.8 thus showing no significant differences between the invaded and the uninvaded plots. This finding is in agreement with the report of Stoll et al. (2012) in Switzerland, but contradictory to the results by Dassonville et al. (2011) who reported significant decrease of soil acidity after *F. japonica* invasion. Rahmonov et al. (2014) reported a high variable pH at sites invaded by *F. japonica*, and *F. japonica* can survive very harsh condition with a pH range of 3.0 to 8.5 (Sołtysiak and Brej 2014; Chmura et al., 2015).

Most previous studies detected a higher abundance, biomass and/or species richness of fungi, and a lower bacterial abundance or biomass in *F. japonica* stands than in the uninvaded sites (Lavoie, 2017). For example, Suseela et al. (2016) recorded up to eight times greater abundance of fungi and 61% lower bacterial biomass under *F. japonica* than in native vegetation attributed to the accumulation of slowly decomposing knotweed litter that favors fungi over bacteria. Stefanowicz et al. (2016) recorded significant decrease of microbial biomass, urease activity, fungal phospholipid fatty acids (PFLA), fungal:bacterial PLFA ratio, gram-negative bacterial PLFA, and soil microbial respiration under *F. japonica* under natural conditions. By contrast, in mesocosm pot experiments carried out by Stefanowicz et al. (2019), *F. japonica* did not reduce microbial activity or biomass but increased fungal biomass and fungal:bacterial ratio. The analyzed microbial characteristics in our study revealed that SMR and MBC content were slightly higher (but not significantly) in all ecosystems invaded by *F. japonica* in comparison to the uninvaded ones, suggesting that invasion did not affect microbial activity. This was in line with the measured enzyme activities, which remained unaffected by *F. japonica* invasion probably due the unchanged soil acidity, a factor affecting activity of soil enzymes (Dassonville et al., 2011).

To the best of our knowledge, nematode communities have never been studied in natural habitats invaded by *F. japonica*. Our study revealed a negative effect of *F. japonica* on nematode communities, with lower nematode abundance and species richness in the invaded than in the uninvaded plots. These results are consistent with those from an analysis of nematode communities in three habitats invaded by *Heracleum sosnowskyi* in central Lithuania (Renčo and Baležentiené, 2011). Lower diversity of plant species in ecosystems may affect the populations of plant-parasitic nematodes (Yeates, 1999). This is related to rich root system of higher plants that serves as a food source for plant nematodes (Bongers, 1990), therefore the assessment of nematode abundance and their species diversity reflects the variations in the nematode community due to changes in plant communities (Viketoft et al., 2005). These reports are in line with our findings of a negative impact of *F. japonica* on the abundance of plant-parasitic and root-fungal feeder nematodes in the invaded plots. Omnivores and predators tend to be more sensitive to environmental changes (Bongers, 1990; Yeates et al., 1993, Ferris et al., 2001) because of their longer generation times and lower fecundity. In our study, the abundance of omnivores was significantly lower in the invaded than in the adjacent uninvaded plots, contradicting the results from a laboratory experiment found by Abgrall et al. (2018), where knotweed rhizome extracts in different concentrations were applied to soil collected in an invasion-prone site. We can only speculate why omnivores in some cases behave to *F. japonica* invasion as typical K-strategist and why not in others (Abgrall’s extracts). It may be that their diverse and often unknown feeding strategies is hampering data interpretation (Cesarz et al., 2015), or the different study conditions (natural vs laboratory). In contrast, differences in predator abundances were not significantly different between the invaded and the non-invaded plots, confirming the findings by Abgrall et al. (2018) and De Deyn et al. (2004) that the change in plant communities, roots diversity, and biomass production do not affect the nematodes of higher trophic groups such as predators.

According to Bongers (1990), the Maturity index (MI) represents the degree of environmental disturbance, with lower values being indicative of a more disturbed and enriched environment, and higher values being characteristic of a less disturbed and stable environment. We observed lower MI only in the invaded compared to the uninvaded plots, thus conforming with results presented by Renčo and Baležentiené (2011) in sites invaded by *H. sosnowskyi* in Central Lithuania. Ferris et al. (2001) proposed the Enrichment index (EI) to be a measure of opportunistic bacterivores and fungivores nematodes; the higher EI values in the invaded plots indicative of an N-enriched, highly disturbed environment with low C:N ratio (Ferris et al., 2001). The Structure Index (SI) is the relative contribution of nematodes with higher cp-value (3–5) and indicates the state of food webs affected by stress or disturbance (Ferris et al., 2001). The value of SI can also specify the possibility of control of predators, but in our study, it did not differ significantly between the invaded and the uninvaded sites or ecosystems, confirming the findings by Renčo et al. (2019) and Čerevková et al. (2019) in ecosystems invaded by *Heracleum sosnowskyi*. Low values of the Channel index CI (<50%) indicates decomposition pathways dominated by bacteria whereas high CI (>50%) indicates a higher proportion of fungal decomposition (Ferris, et al., 2001). In the present study, significant differences in CI were found between the invaded and the uninvaded plots, but all observed value was lower than 50% indicating bacterial-dominated decomposition.

In conclusion, our study demonstrated that the invasive plant species *F. japonica* considerably negatively altered nematode communities (total abundance, species composition, the abundance of trophic groups) in all habitats. Surprisingly, *F. japonica* invasion negatively affected neither soil moisture nor soil acidity, and neither SMR and MBC content nor soil enzymes activity. But, there is some doubt that the abundant litter produced and the deep rhizome system of invasive *F. japonica* have an overall negative impact on soil moisture and pH to be the benefit of the invader, supported by our findings. Most of the studies performed were, however, local, including ours from three habitats. The extent of impact *F. japonica* invasion has on soil nematode communities, as well as microbial characteristics on the regional to national scale under a variety of natural conditions remains to be verified in future studies.
